# Association of endogenous circulating sex steroids and condition-specific quality of life domains in postmenopausal women with pelvic floor disorders

**DOI:** 10.1007/s00404-018-4650-7

**Published:** 2018-01-15

**Authors:** Barbara Bodner-Adler, Klaus Bodner, Oliver Kimberger, Ksenia Halpern, Heinz Koelbl, Wolfgang Umek

**Affiliations:** 1 Department of General Gynecology and Gynecologic Oncology, Medical University of Vienna, Währinger Gürtel 18-20, 1090 Vienna, Austria; 2 Department of Anesthesiology, Medical University of Vienna, Vienna, Austria; 3 Outcomes Research Consortium, Cleveland, OH USA; 4 Department of Special Gynecology and Obstetrics, Karl Landsteiner Institute, Vienna, Austria

**Keywords:** Health-related quality of life, Pelvic floor questionnaire, Pelvic organ prolapse, Stress urinary incontinence, Endogenous sex steroids, Postmenopausal women

## Abstract

**Objective:**

To examine the relationship between endogenous sex steroids and various condition-specific quality of life domains in postmenopausal women with pelvic floor disorders. We hypothesized that woman with lowest androgen and estradiol concentrations would report worse scores of quality of life domains.

**Methods:**

Forty-six women with pelvic organ prolapse (POP) and 47 cases with stress urinary incontinence (SUI) answered the validated pelvic floor questionnaire and underwent serum sex steroid measurement. A multivariate logistic regression model was used to determine the association between subjective outcome parameters and serum hormonal levels after adjusting for confounders.

**Results:**

Univariate analysis revealed a strong inverse correlation between serum estradiol level (E2) and prolapse domain score (correlation coefficient = 0.005) as well as a significant positive correlation between SHBG level and prolapse domain score (correlation coefficient = 0.019) in cases with POP. Furthermore, the sex domain score showed a significant negative correlation with the androstendion (correlation coefficient = 0.020), DHEAS (correlation coefficient = 0.046) and testosterone level (correlation coefficient = 0.032) in the POP group. In the multivariate model, high serum SHBG (CI: 0.007–0.046) remained independently associated with worse scores in the prolapse domain and low serum DHEAS (CI: − 0.989 to 1.320) persisted as a significant predictor for a worse score in the sex domain. Regarding SUI cases, no association was noted between serum hormonal levels and quality of life related pelvic floor domains (correlation coefficient > 0.05).

**Conclusion:**

Our results suggest that pelvic floor related quality of life might also be affected by endogenous sex steroids in POP, but not in SUI cases.

## Introduction

Pelvic floor disorders (PFD) are common gynecologic complaints that adversely affect the quality of life of many women because of their impact on medical, social, emotional, and sexual well-being [1]. Validated questionnaires and scales play an important role in identifying symptoms of a disease, helping clinicians assess patient’s quality of life [2]. They are reliable and valid instruments of subjective outcomes and are recommended to use in clinical as well as research settings [3]. Furthermore, the levels of sex steroids, particularly estrogens and androgens, change markedly during menopause [4] and the risk to develop a pelvic floor disorder of any kind increases significantly in postmenopause. It is well known that estrogen deficiency after menopause causes atrophic changes of the urogenital tract and is also associated with various urinary symptoms. Furthermore, estrogen levels have a significant meaning in the function of the genital and lower urinary tract, as there is evidence of the presence of estradiol receptors in the urinary bladder, urethra, vagina and pelvic floor musculature [5]. Female stress urinary incontinence (SUI), the most common type of incontinence, is defined as an complaint of involuntary loss of urine on effort or physical exertion, or on sneezing or coughing [6, 7]. There is epidemiological evidence from several studies implicating menopause and subsequent estrogen deficiency in the pathogenesis of a number of urinary complaints including incontinence. Besides, the prevalence of prolapse increases in the postmenopausal period, suggesting that a hypoestrogenic state is an important contributing factor [8, 9]. Additionally, these disorders can also have a detrimental impact on health-related quality of life and women’s sexual well-being [10]. For instance, several studies reported that women with POP may experience lower libido, are less likely to be sexually active compared to women without POP [11]. The association between circulating endogenous sex steroids and condition-specific quality of life domains has not been investigated so far among women with PFD.

The aim of the present study was to determine whether a relationship exists between serum steroid hormones and various condition-specific pelvic floor domains in postmenopausal women with SUI or symptomatic POP. Because epidemiologic evidence suggests that declined hormonal levels increase PFD, we hypothesize that endogenous hormonal levels might also have an impact on quality of life related pelvic floor domains.

## Materials and methods

### Comprehensive Pelvic Floor Questionnaire

All patients with symptomatic pelvic organ prolapse or verified SUI received the German Version of the pelvic floor questionnaire (return rate: 96% in all) to investigate the relationship between lower urinary tract symptoms and serum hormonal levels. Questionnaires were filled out at the urogynecologic outpatient clinic after physical examination and blood collection. Participants ran through the questionnaire by themselves, in case of any difficulties a nurse could assist. The German Pelvic Floor Questionnaire is a self-administered, validated questionnaire, which integrates bladder, bowel and sexual function, pelvic organ prolapse, severity, bothersomeness and condition-specific quality of life in women with urinary incontinence (UI) and/or POP. The questionnaire is divided into four domains (bladder, bowel, pelvic organ prolapse, sexual function) and each question is scored from zero to four. The additive scores are divided by the maximum reachable score and multiplied by ten, giving a value between zero (0 = no symptoms) and 10 for each of the domains. Results of the validation study and scoring system have been previously published by Baessler et al. [12].

### Study population

As published previously, women were recruited from the urogynecologic outpatient clinic at the Medical University of Vienna (MUV, Austria) between 2015 and 2016 and the study was approved by the ethics committee of Medical University Vienna (EK no. 2174/2015) [13, 14]. All participants gave written, informed consent and the main outcome variable of interest at this time was subjective health-related quality of life.

Women included in this study were postmenopausal, able to read and write and understand German, and were seeking treatment either for SUI or POP (≥ POP-Q stage II). Women were excluded if menopausal status was unclear, if the diagnosis included urgency incontinence (defined as a complaint of involuntary loss of urine associated with urgency), mixed urinary incontinence (defined as a complaint of involuntary loss of urine associated with urgency and also with effort or physical exertion or on sneezing or coughing) or urgency (defined as complaint of a sudden, compelling desire to pass urine which is difficult to defer), cancer, intake of systemic or local hormonal treatment or intake of selective estrogen receptor modulators, severe endometriosis or pelvic inflammatory diseases.

After consent, women underwent physical and pelvic examination, including a standardized urogynecologic interview, complete physical examination to check for genital prolapse according to ICS POP-Q-system [15] and controlled provocation with 300 ml saline in the bladder according to the International Continence Society (ICS) [16]. SUI was tested and verified using a cough test and a pad test (quantification of the amount of urine lost over the duration of 1 h, by measuring the increase in the weight in the perineal pads used) with a standardized bladder filling [17]. Urodynamics were not performed.

### Assay methods

Furthermore, blood samples were withdrawn from all participants for the in vitro quantitative determination of circulating sex steroids (Electrochemiluminescence immunoassay ECLIA). Serum concentrations of Follicle-stimulating hormone [FSH], luteinizing hormone [LH], 17β-estradiol [E2], sex hormone binding globulin [SHBG], testosterone [T], dehydroepiandrosterone sulfate [DHEAS] and androstendion [AEON] were determined by immunoassay using commercial kits according to the manufacturer’s protocols (elecsys®2010 Systems, Roche Diagnostics International, Switzerland). Analytic sensitivity and the coefficient of variation (CV%) were as follows: for T 0.02 ng/ml and CV 4–8%; for SHBG 0.5 nmol/L and CV 5%; for E2 10 pg/ml and CV 4–8%; for AEON 0.3 ng/ml and CV 5–7%, for FSH 0.1 mU/ml and CV 7%; for LH 0.1 mU/ml and CV 4%; for DHEAS 0.1 μg/dl; CV 5–8% and for PRL 1.00 ng/ml; CV 4–7%, respectively.

Women were classified into two groups: patients with verified SUI (*n* = 47) and patients with symptomatic POP (*n* = 46). Clinical information, including follow-up data was obtained from the database of the department of General Gynecology and Gynecologic Oncology. All patient records were anonymized and de-identified prior to analysis.

### Statistical analysis

Chi-square was used for the comparison of categorical variables between the two groups and Student’s *t* test for continuous variables. The average score of each domain in the questionnaire was reported as mean and standard deviation. For correlation analysis Spearman test was used with correlation coefficient. Multivariate stepwise logistic regression (including backward elimination) was performed to identify parameters associated with quality of life related pelvic floor domains. A *p* value < 0.05 was considered statistically significant. The SPSS system (IBM, Armonk, NY, USA, Version 23) was used for the calculations.

## Results

Ninety-seven women in all gave consent to participate in this study, 93 of whom (96%) were finally included after returning an evaluable complete survey [46 with symptomatic pelvic organ prolapse (POP) ≥ stage 2 and 47 with verified SUI]. Missing data did not exceed 4%. In our study population, 65 out of 93 (70%) reported sexual activity with sexual intercourse. Clinical characteristics of all study participants are shown in Table 1. No statistically significant differences could be observed between the two groups. Mean scores of various quality of life related pelvic floor domains are presented in Table 2.

**Table 1:** Mean values (SD) of patients characteristics in women with SUI and symptomatic POP

Parameter	SUI *n* = 47	POP *n* = 46	*p* value
	*n* (%) or mean (± SD)	*n* (%) or mean (± SD)	
Age (years)	63 (± 11.1)	65 (± 9.9)	> 0.05
Menopause age (years)	52 (± 2.9)	52 (± 2.3)	> 0.05
Years from menopause	14 (± 9.6)	13 (± 9.4)	> 0.05
BMI (kg/m^2^)	27.3 (± 5.2)	27.70 (± 5.7)	> 0.05
Hypertension	23 (50%)	14 (30%)	> 0.05
Smoking	14 (30%)	10 (22%)	> 0.05
Parity	2.2 (± 1.9)	2.4 (± 1.7)	> 0.05
Mode of delivery			> 0.05
SVD	45 (96%)	42 (91%)	
Vaginal-operative	2 (4%)		3 (7%)
Cesarean section	0 (0%)	1 (2%)	
Profession			> 0.05
Sitting	16 (34%)	13 (28%)	
Physical heavy	14 (30%)	19 (42%)	
Others	17 (36%)	14 (30%)	

*SD* standard deviation, *SVD* spontaneous vaginal delivery

**Table 2:** Mean values (SD) of pelvic floor related quality of life domains in cases with SUI and cases with POP

Domains	SUI = 47	POP = 46	*p* value
	Mean (± SD)	Mean (± SD)	
Bladder	4.32 (± 1.49)	2.85 (± 1.59)	0.000*
Bowel	2.64 (± 1.29)	2.64 (± 1.54)	> 0.05
Prolapse	1.09 (± 1.09)	4.43 (± 2.29)	0.000 *
Sexual function	2.69 (± 1.95)	2.59 (± 2.03)	> 0.05
In all	2.57 (± 1.09)	3.24 (± 1.30)	0.009 *

*SD* standard deviation

*Significant; *p* < 0–05

### Relationship between sex steroid serum levels and quality of life domains in cases with symptomatic POP

Univariate analysis revealed a statistically significant inverse correlation between E2 level and prolapse domain score (correlation coefficient = 0.005), signifying more symptom bother from prolapse in women with low E2 levels as well as a significant positive correlation between SHBG level and prolapse domain score (correlation coefficient = 0.0019). Furthermore, the sex domain score showed a significant negative correlation with androstendion (correlation coefficient = 0.020), testosterone (correlation coefficient = 0.032) as well as with DHEAS level (correlation coefficient = 0.046), demonstrating that women with low androgen concentration had significantly higher sex domain scores. No statistically significant correlations could be observed between serum hormonal levels and mean bladder, and bowel score (correlation coefficient > 0.05). Furthermore, no correlation between POP-Q stage and prolapse domain score was detected (> 0.05).

### Relationship between sex steroid serum levels and quality of life domains in cases with SUI

No statistically significant correlation could be observed between serum hormonal levels and mean bladder; sex, bowel and prolapse score (correlation coefficient > 0.05). Moreover, no correlation between degree of SUI and bladder domain score could be observed (*p* > 0.05). A significant positive correlation was detected between the bladder domain score and the sex domain score (correlation coefficient = 0.008) (Table 3).

**Table 3:** Multivariate logistic regression analysis with the domain prolapse as the dependent variable and clinical characteristics and hormonal values as independent variables

Parameter	OR	95% CI	*p* value
Age	0.011	− 0.067 to 0.089	0.870
Menopausal age	− 0.001	− 0.378 to 0.259	0.750
BMI	− 0.001	− 0.118 to 0.116	0.983
Smoking	− 3.3337	− 4.835 to − 1.839	0.000*
Parity	0.242	− 0.160 to 0.645	0.046
Testosterone	− 8.449	− 17.480 to 0.582	0.066
DHEAS	0.211	− 0.989 to 1.320	0.701
Androstendion	0.194	− 1.443 to 1.831	0.811
SHBG	0.027	0.007 to 0.046	0.009*
E2	− 0.034	− 0.100 to 0.033	0.307
POP-Q stage	− 0.079	− 1.146 to 0.989	0.882

*OR* odds ratio, *CI* confidence interval

*Statistically significant

### Multivariate analysis in cases with POP

When controlled for age, parity, menopausal age, BMI, smoking and various hormonal parameters through multivariate logistic regression, the relation between E2 level and prolapse domain score was no longer significant, but SHBG level remained an independent predictor for worse prolapse domain score (OR and CI: 0.027 and 0.007–0.046) and DHEAS level was independently negatively related to a worse sex domain score (OR and CI: − 1.444 and − 2.763 to − 0.126) (Table 4).

**Table 4:** Multivariate logistic regression analysis with the domain sexual function as the dependent variable and clinical characteristics and hormonal values as independent variables

Parameter	OR	95% CI	*p* value
Age	− 0.158	− 0.260 to − 0.056	0.004*
Menopausal age	0.176	− 0.186 to 0.538	0.328
BMI	0.077	− 0.067 to 0.222	0.282
Smoking	1.539	− 0.130 to 3.208	0.069
Parity	0.188	− 0.258 to 0.634	0.396
Testosterone	4.171	− 6.348 to 14.689	0.424
DHEAS	− 1.444	− 2.763 to − 0.126	0.033*
Androstendion	− 0.548	− 2.361 to 1.285	0.551
SHBG	− 0.027	− 0.057 to 0.004	0.084
E2	0.287	0.023 to 0.550	0.034*
POP-Q stage	0.471	− 0.737 to 1.679	0.432

*OR* odds ratio, *CI* confidence interval

*Statistically significant

## Discussion

Urinary incontinence and pelvic organ prolapse affects a great portion of postmenopausal women and are a major cause of quality of life impairment [7]. The levels of sex steroids, particularly estrogens and androgens, change markedly during menopause and estrogen deficiency after menopause causes atrophic changes of the urogenital tract and is also associated with symptoms of the lower urinary tract system [18]. However, the association between circulating endogenous sex steroids and condition-specific quality of life domains has not been investigated so far within this population. We have examined the relationship between circulating sex steroids and various self-reported condition-specific pelvic floor domains in postmenopausal women with SUI or symptomatic POP.

### Main findings

Our data imply that in a sample of postmenopausal prolapse patients, circulating androgens are strongly related to prolapse and sex domain scores, as high serum SHBG remains independently associated with a worse score in the prolapse domain and low serum DHEAS persists as a significant predictor for a worse score in the sex domain. Our data do not support the notion, that androgens and/or estrogens exert an influence on quality of life related pelvic floor domains in cases with SUI.

### Comparison with literature

Several studies showed that women diagnosed with PFD are more likely to report quality of life impairment and sexual difficulty related to pelvic floor symptoms, including sexual avoidance due to vaginal prolapse or sexual activity restriction due to fear of urinary incontinence [7, 18]. Furthermore, very few reports have investigated the relationship between serum hormonal levels and incontinence complaints. For instance, one study examined the relationship between serum testosterone level and self-reported female urinary incontinence. The authors reported that very low serum testosterone levels were associated with an increased likelihood of stress and mixed urinary incontinence [19]. Another study examined a large population of perimenopausal women and found no relationship between serum testosterone and incontinence [20]. Augoulea et al., who assessed a number of endogenous hormonal levels in 275 perimenopausal women with and without SUI, found that healthy women had higher serum levels of estrogen and androstendion compared to women with SUI [7]. Besides, a number of studies have shown the presence of androgen receptors in the female pelvic floor as well as the anabolic effect of androgens on the pelvic floor [21].

In our study, the main outcome of interest was a possible association between subjective pelvic floor related quality of life and hormonal levels. Our results showed no association between hormonal levels and subjective outcome parameters in cases with SUI, but we could detect an association between hormonal levels and subjective outcome parameters in prolapse patients. In our study high SHBG concentration was independently associated with a worse score in the prolapse domain and low DHEAS concentration was significantly negatively related to a worse score in the sex domain. SHBG is best known as a carrier protein for circulating E2 and T and its production is under the physiological control of E2, which positively regulates SHBG, testosterone and other androgens, which negatively regulate SHBG [22]. Higher SHBG levels are generally associated with lower levels of bioactive fractions of sex steroids. It is unclear so far, whether or not SHBG itself is capable of transducing intracellular signals [23]. The association between SHBG and prolapse domain score is likely due to one important factor of reproductive hormones (androgens and estrogens). The inverse relationship between DHEAS and sex domain score suggests that androgens of adrenal origin might influence sexual well-being in females. In some clinical trials DHEA administration also improves the sense of well-being of peri-and postmenopausal women [24].

To our best knowledge, there are no studies that have examined sex steroids and self-reported quality of life domains among postmenopausal women with PFD although several quality of life domains have been related to other factors like BMI, metabolic syndrome and so on. In our opinion, subjective outcome is of great importance and it is necessary to look on all predictors, which might have a detrimental effect on women’s quality of life and sexual well-being. For this purpose, condition-specific quality of life instruments were developed. Interestingly, our data demonstrated no association between POP-Q stage and subjective prolapse domain scores, implicating that a severe prolapse stage must not be mandatory associated with strong psychological strain and contrariwise. In our opinion this underlines the fact that it is necessary to consider not only the underlying anatomical disorder, but also women’s overall pelvic function and their health-related quality of life when making treatment decision.

### Limitations of the study

We are aware of the limitations of our study. This was a retrospective secondary analysis of a study, which primarily aimed at studying the relationship between circulation sex steroids in women and their symptom severity. This limits the possibility to study cause and effect relationship. Thus, the authors can only comment on associations between hormonal levels and health-related quality of life scores, but not on the underlying causality. On the other hand this is the first clinical study investigating the relationship between subjective outcome parameters like various quality of life related pelvic floor domains and circulating and sex steroid levels in a sample of postmenopausal women with PFD. However, future prospective studies are still needed.

### Summary

Regarding cases with POP, high SHBG level was independently associated with worse score in the prolapse domain and low DHEAS level remained a predictor for worse sex domain score. This might indicate that endogenous sex steroids, like DHEAS and SHBG, can also have a detrimental effect on quality of life in prolapse cases. Subjective outcome is of great importance and it is necessary to look on all predictors, which might have a detrimental effect on women’s quality of life and sexual well-being. Future studies will be needed to confirm our results and evaluate also the potential therapeutic implications to improve quality of life.
